# Glioma-induced neural functional remodeling in the hand motor cortex: precise mapping with ECoG grids during awake craniotomy

**DOI:** 10.1097/JS9.0000000000002277

**Published:** 2025-02-04

**Authors:** Tao Chang, Yihan Wu, Yuxin Quan, Siliang Chen, Jiawei Liao, Wenyu Zhao, Yu Li, Yuan Fang, Yixuan Zong, Yanhui Liu, Ning Jiang, Qing Mao, Jiayuan He, Yuan Yang

**Affiliations:** a Department of Neurosurgery, West China Hospital, Sichuan University, Chengdu, China; b National Clinical Research Center for Geriatric, West China Hospital, Sichuan University, Chengdu, Sichuan, China; c Med-X Center for Manufacturing, Sichuan University, Chengdu, Sichuan, China; d Department of Anesthesiology, West China Hospital of Sichuan University, Chengdu, Sichuan, China

**Keywords:** direct cortical stimulation, electrocorticography, functional mapping, glioma, motor cortex

## Abstract

**Background:**

The dilemma of achieving ‘onco-functional balance’ in gliomas affecting the motor cortex highlights the importance of functionally-guided resection strategies. While accurate mapping of eloquent areas often requires frequent electrical stimulation, this practice can lead to side effects like seizures and postoperative deficits. To enhance safety in functional mapping, we studied how gliomas impact hand movement areas and assessed the effectiveness of cortical electrical activity for functional mapping in this setting.

**Materials and methods:**

We recruited patients with gliomas affecting the motor cortex and individuals with an unaffected motor cortex for awake craniotomy. During the procedures, electrocorticography (ECoG) grids were employed to record signals under three conditions: resting state, finger movements, and wrist movements. We then quantified the distances from the positively stimulated sites to the specific anatomical landmarks. Additionally, we analyzed the relationship between the ECoG power features and the stimulation responses.

**Results:**

The cortical layout for finger activity in the motor cortex glioma (MCG) group was more dispersed and overlapped, typically clustering near the central sulcus and Sylvian fissure. The predictive performance of ECoG mapping exhibited significant variability across different frequency bands and clinical scenarios. Specifically, the area under the curve (AUC) for the non-MCG group during the resting state reached its peak, with a value of 0.802 for Gamma3 (95% CI = 0.729–0.875) and 0.865 for broadband (95% CI = 0.804–0.926). In contrast, the MCG group achieved the highest AUC during wrist movements, with Gamma3 at 0.785 (95% CI = 0.719–0.849) and broadband at 0.824 (95% CI = 0.753–0.890).

**Conclusion:**

Gliomas in the motor cortex disrupt the distribution of hand activity, complicating intraoperative functional mapping. As a novel and reliable approach, ECoG technique can complement and guide direct cortical stimulation for precise mapping, potentially reducing its frequency, minimizing the risk of functional deficits, and achieving a balance between maximal tumor resection and neurological preservation.

## Introduction

Approximately, 25% of low-grade gliomas and 10% of high-grade gliomas occur in the motor cortex, including the supplementary motor area (SMA), premotor cortex (PMC), and primary motor cortex (M1)^[1]^. Balancing maximal resection with the preservation of neurological function is essential, as this approach can improve overall survival, minimize postoperative neurological impairments, and enhance the quality of life^[2-4]^. The dilemma of achieving ‘onco-functional balance’ in motor cortex gliomas highlights the pivotal role of functionally guided mapping resection strategies^[5,6]^.

Currently, awake craniotomy (AC) with direct cortical stimulation (DCS) is considered the best strategy for achieving these surgical objectives, as it minimizes injury to functional areas and subcortical white matter tracts^[7,8]^. DCS, a stimulation technique that induces transient functional impairment by directly evoking cortical activity, is recognized as the gold standard for mapping the eloquent cortex^[4,9,10]^. However, this procedure may not be feasible for specific populations, as highly heterogeneous intraoperative conditions are influenced by factors such as preoperative cognitive status, age (<15 or >65 years), pain, and anesthesia^[11]^. Additionally, excessive intensity can provoke seizures, while repeated high-frequency activation in the same area may lead to postoperative functional deficits^[12]^.

Traditional approaches have emphasized a discrete, orderly distribution of hand activities, as suggested by historical “homunculus” depictions^[13]^. However, functional magnetic resonance imaging (MRI) studies in healthy individuals revealed substantial overlap in the cortical areas activated by different body parts^[14,15]^. Despite this intersection, the regions responsible for specific finger movements remained relatively consistent across individuals. In clinical practice, gliomas in the motor cortex can reshape the morphology of the hand motor area and alter neural population dynamics, thereby offering insights into the structural reorganization and functional plasticity^[16]^. Nonetheless, the disruption of somatotopic organization of hand representations caused by this condition remains elusive, further complicating intraoperative stimulation mapping.

Electrocorticographic (ECoG) activity fluctuations associated with specific stimuli or cognitive tasks have been utilized for functional mapping^[17-20]^. Previous studies have suggested that neural oscillations within various rhythms are involved in a wide range of cognitive processes by inducing synchronization across specialized brain regions^[21]^. Mu and beta frequencies, typically generated by thalamocortical circuits, decrease in amplitude during actual or imagined movements and exhibit broad spatial distributions^[22,23]^. In contrast, gamma activity (above 30 Hz), believed to be produced by local cortical circuits, increases focally with cortical activation and is used in offline brain mapping^[24-26]^. Vansteensel et al^[27]^, examined epilepsy patients with ECoG recording and DCS during spontaneous hand movements and resting state, finding high specificity (>0.80) across all frequency bands, with the 65–95 Hz showing a sensitivity of 0.82 in detecting stimulation-positive sites. Nonetheless, there is a paucity of research employing the ECoG technique to delineate the hand representation region within motor cortex gliomas, with even fewer studies examining this complexity across various neural activity states. This research represents an initial venture toward identifying the eloquence cortex based on neural dynamics rather than architectural markers in motor cortex gliomas.

We explored the altered distribution of hand-specific activity in motor cortex gliomas. Furthermore, by capturing cortical signals through ECoG grids during AC, this innovative technique enabled precise mapping of the hand motor cortex. And, its predictive performance showed significant variability across different frequency bands and task states. Therefore, this approach serves as an effective complement and guide to DCS, potentially reducing the frequency of stimulation, minimizing the risk of functional deficits, and achieving a balance between maximal tumor resection and neurological preservation.

## Materials and methods

### Participants, consent, and ethics approval

**Figure 1.**
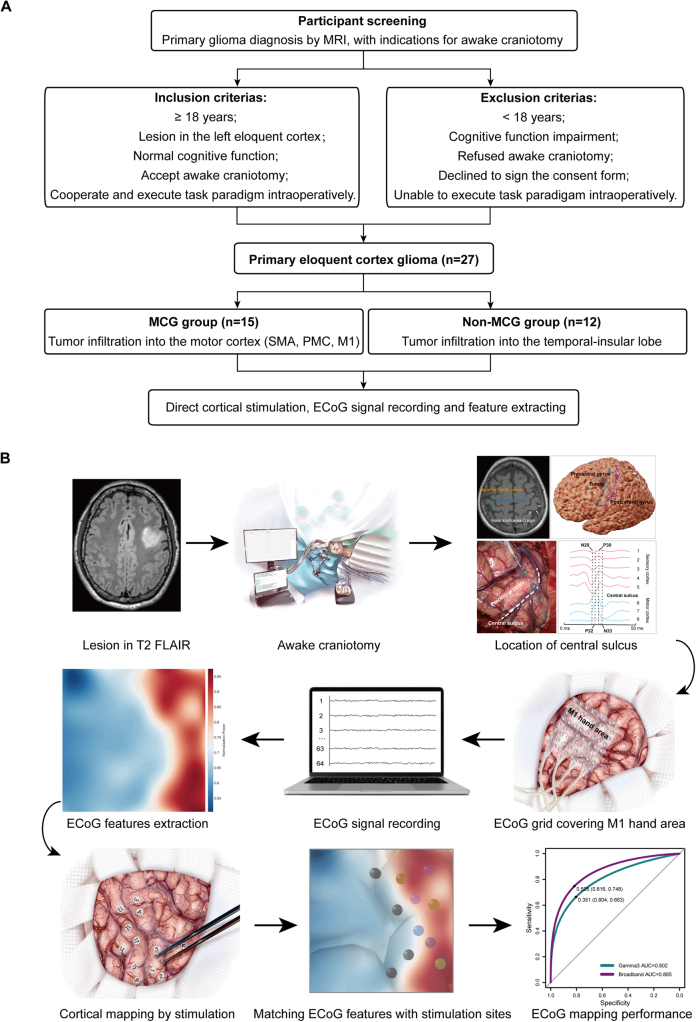
Flowchart and key steps for this research. (A) Flow diagram for participant screening and grouping. (B) The main steps of this study include: selecting patients for AC; employing multimodal approaches to guide the placement of ECoG grid over the M1 hand area; recording ECoG signals; offline signal feature extraction; cortical stimulation mapping; matching ECoG mapping with stimulation sites; evaluation the performance of motor cortex mapping by ECoG power feature.

All participants with primary diffuse gliomas were recruited from our department. The inclusion criteria were as follows: ages 18–65, left-sided lesions in the temporal-insular lobe or motor cortex, no epileptiform discharges on presurgical EEG (Neuroelectrics, Starstim32® tES-EEG Systems, Austria), no psychiatric or neurological disorders, no substance abuse, no severe motor dysfunction, ability to perform motor tasks during awake surgery, and diagnosis of primary diffuse gliomas according to the 2021 WHO classification guidelines^[14]^. Exclusion criteria included any neuropsychiatric disorders, other central nervous system comorbidities, prior treatments with stereotactic radiotherapy, chemotherapy, or other surgeries, and the presence of other cerebral tumors (Fig. 1).

### Movement task paradigm

During the surgery, a 15-inch laptop computer running a custom Python script (Version 3.7) with a 60 Hz refresh rate was positioned 30 cm away from the patient’s face. Upon awakening from anesthesia, patients were subsequently instructed to perform tasks in response to cues provided by the video on the screen. The paradigm included five sequential sessions, each lasting approximately 60 seconds, interspersed with 3-second rest periods between sessions. Each session consisted of six trials, with each trial involving specific finger or wrist flexion-holding-extension (Supplementary Figure 1, http://links.lww.com/JS9/D903). To minimize noise in the operating room, all personnel were instructed to refrain from speaking, and devices such as telephones, machinery alarms, surgical suction, and other nonessential equipment were either muted or temporarily shut down.

### AC, electrodes localization, and ECoG signal acquisition

The patient’s consciousness was managed through an asleep–awake–asleep procedure, during which a laryngeal mask was used for intubation in the asleep phases. After the patient regained consciousness, anesthesia administration and evaluation were conducted to investigate the feasibility of ECoG signal recording. The following approaches were implemented^[28]^: (1) Propofol was discontinued 10–15 minutes prior to signal recording. (2) During this awake phase, an infusion of remifentanil at 0.01–0.025 μg/(kg·min) was maintained to relieve the patient’s pain and discomfort without significantly affecting respiration or hemodynamics. (3) The patient remained sedated but was able to respond to questions and follow instructions accurately. (4) The level of sedation was assessed by the Observer’s Assessment of Alertness/Sedation Scale, targeting a score of ≥4 (where 1 indicates general anesthesia and 5 denotes full alertness) and a Bispectral Index score > 80, indicating an awake state.

All participants initially underwent intraoperative multimodal mapping of the central sulcus, including MRI, neuro-navigation systems (BrainLab AG, Munich, Germany), and neurophysiological monitoring (MNE2000, Nihonkohden, Japan). Platinum arrays (HKHS Healthcare Co., Ltd., Beijing, China) were embedded in a plastic sheet, with each electrode featuring a contact surface diameter of 2 and 5 mm spacing between electrodes, typically configured in an 8 × 8 array. Needle electrodes placed on the ipsilateral and contralateral scalps served as the ground and reference, respectively. Signals were recorded using the NeuSen H (Neuracle Technology Co., Ltd., Changzhou, China), which amplified and sampled data at rates ranging from 1 to 2 kHz. A 2-minute resting-state ECoG signal was initially collected, followed by recordings while the patient performed finger and wrist tasks.

By integrating stereotactic neuro-navigation, intraoperative photographs of the cortex with overlaying grids, and anatomical landmarks from MRI, each electrode was registered to either the MRI T2-weighted fluid-attenuated inversion recovery or T1 post-gadolinium images using MRICro software (http://www.mccauslandcenter.sc.edu/mricro/). Subsequently, ECoG grids were projected onto a standardized brain model, the Montreal Neurological Institute (MNI) template space, with a 0.8 mm isotropic volumetric map, and visualized by Brainstorm software (https://neuroimage.usc.edu/brainstorm/) for each participant. These mappings were reviewed and confirmed by the study’s principal investigators for consensus, while the electrophysiological specialist independently processed the data, remaining blinded to the results.

### Cortical stimulation mapping

All participants underwent DCS for mapping using a bipolar stimulator (Nicolet Cortical Stimulator, Natus Medical Incorporated, Middleton, WI, USA) after the ECoG recording. The settings included a 5 mm interval, 60 Hz frequency, and 1 ms pulse width, with stimulation currents ranging from 1.0 to 5.0 mA, lasting for 2 to 5 s^[4]^. A positive response, evidenced by induced movement in the contralateral limbs, was marked with a 3 mm radius marker. Each site was stimulated three times, with at least two positive responses required. When cortical stimulation simultaneously induced movement in multiple fingers, the site was identified as the functional region for all fingers involved. Stimulation sites were also projected onto the MNI template based on their cortical positions (Supplementary Figure 2. link: http://links.lww.com/JS9/D903).

**Figure 2.**
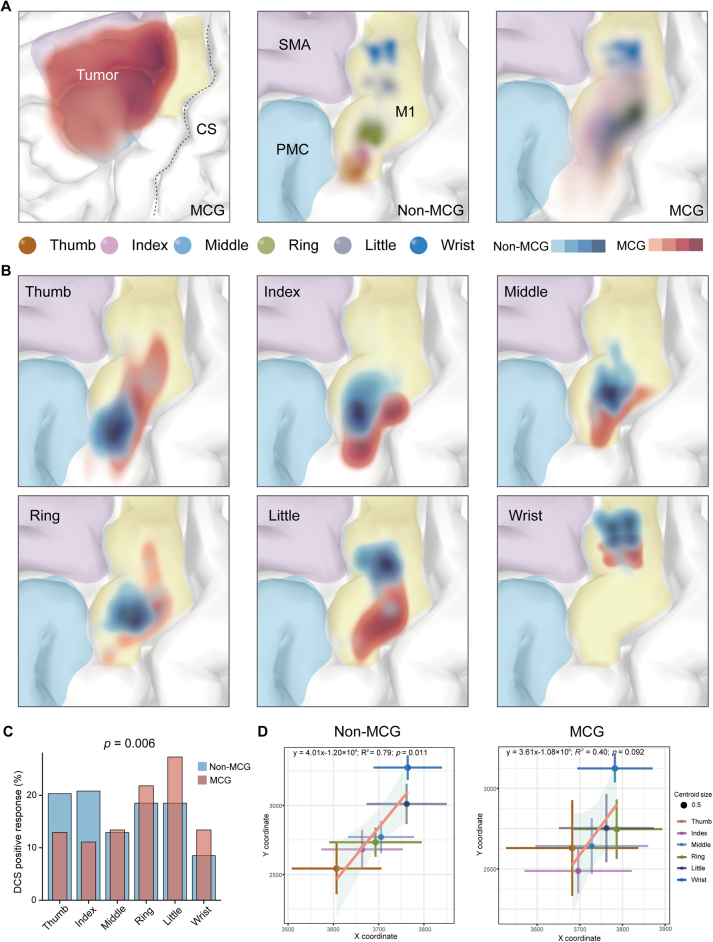
Motor cortex glioma disrupts spatial distribution of hand representation. The lesions in the MCG group primarily infiltrated the SMA, PMC, and M1 cortex (shown in red). A deeper shade of red indicates a higher number of participants with lesions affecting these regions (A, left). In the non-MCG group, specific locations for finger and wrist movements induced by stimulation are identified in the density map (A, middle). In contrast, the cortical representations in the MCG group display a significantly disordered spatial arrangement (A, right). (B) Comparing cortical activation for the same finger between the two groups, the distribution is distinct, predominantly clustering near the central sulcus and Sylvian fissure, while being situated farther from the precentral sulcus and sagittal fissure in the MCG group. (C) Movements in the thumb and index fingers are more commonly induced in the non-MCG group, whereas the MCG group more frequently triggers movements in the ring and little fingers. (D) The distribution of centroids for passive movements in MNI coordinates exhibits a typical structural organization that aligns with the “homunculus” model in the density map. In contrast, the MCG group displays disrupted effector-specific hand areas due to lesions infiltrating the motor-related cortex.

**Figure 3.**
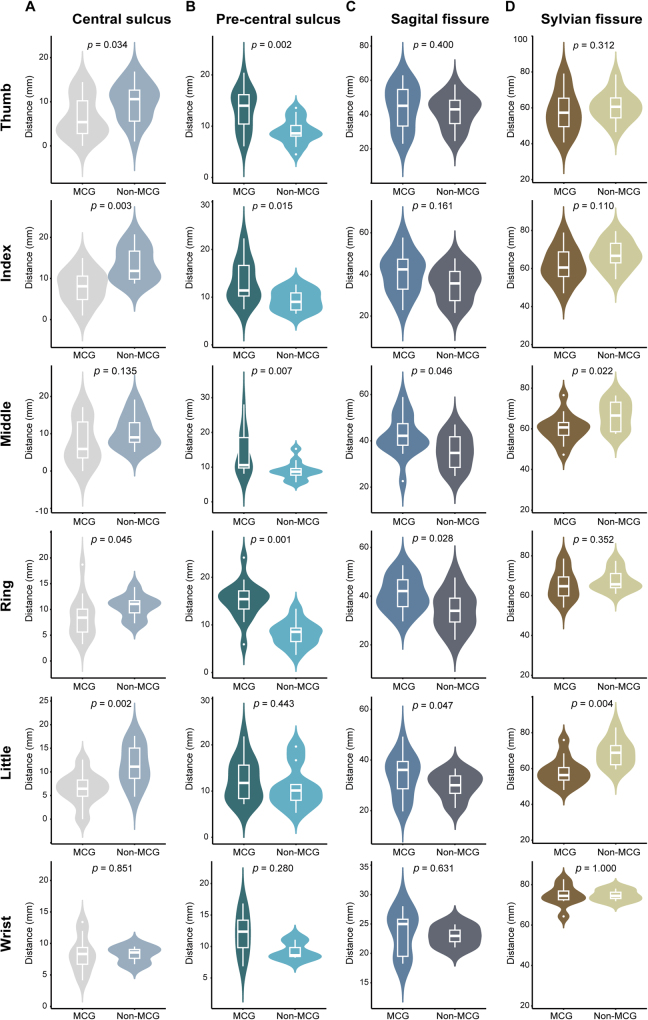
Quantitative analysis of the disruption of hand movement representation by motor cortex glioma. By measuring the distances from positive stimulation sites to anatomical landmarks (including the precentral sulcus, central sulcus, Sylvian fissure, and sagittal fissure), panels A and B reveal that, compared to the non-MCG group, the MCG group’s positive sites are situated closer to the central sulcus and farther from the precentral sulcus. Panels C and D demonstrate that, in the MCG group, these sites are located farther from the sagittal fissure and nearer the Sylvian fissure.

To investigate the spatial distribution of the hand representation region, a kernel density estimation was applied to render these positive sites within the primary motor cortex (R Foundation, Vienna, Austria, version 4.1.2) (Fig. 2). Additionally, the relative distance of each positive site to anatomical landmarks was measured by the minimal Euclidean distance: horizontally to the precentral and central sulci, and vertically to the sagittal and Sylvian fissures (Fig. 3) (Supplementary Figure 3. link: http://links.lww.com/JS9/D903).

### ECoG signal processing

The ECoG signals processing followed several crucial steps. First, a notch filter eliminated power line interference at 48–52 Hz and its harmonics at 98–102 Hz. A FIR bandpass filter was applied to extract signals in the 4–140 Hz range. Channels with excessive noise were excluded from the signal analysis, and any segments with severe noise were preprocessed through global mean interpolation to maintain signal quality. Common average re-referencing was performed to remove common-mode noise artifacts. The average power of each channel in broadband (4–140 Hz) and Gamma3 (80–120 Hz) was computed for finger and wrist movements, as outlined in Equation (4):

$$Power=10*\log_{10}\frac{1}{T}\sum_{t-1}^{T}|f\left(t\right){|}^{2}$$

$$Normalizedpower=\frac{X-X_{min}}{X_{max}-X_{min}}$$

where *f* (*t*) signifies the signal of a specific frequency band, and *T* represents the total number of sampling points during the execution period.

### The relationship between the ECoG power and the stimulation responses

**Figure 4.**
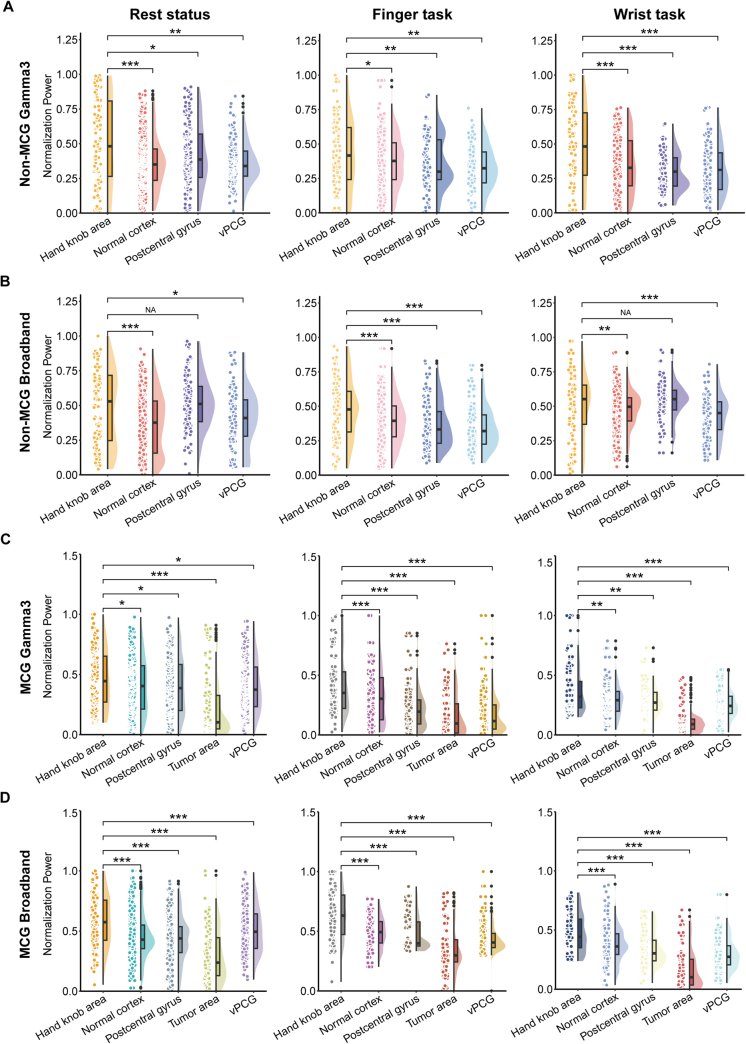
Disparities in ECoG power features across different cerebral regions under various signal conditions. The results show that ECoG power in the M1 hand area is significantly higher than in other cerebral regions.

When cortical stimulation elicited positive movement, the corresponding area was identified as a circle with a 10 mm radius centered on the positive sites^[29]^. Channels within this circle were defined as stimulation-positive electrodes^[30,31]^. The same methodology was applied to analyze the relationship between negative sites and electrodes. We then extracted the ECoG power for each channel and compared the differences between positive and negative electrodes (Supplementary Figure 4. link: http://links.lww.com/JS9/D903). Additionally, we examined the power variations across different regions to assess the feasibility of electrical activity for mapping (Fig. 4).

**Figure 5.**
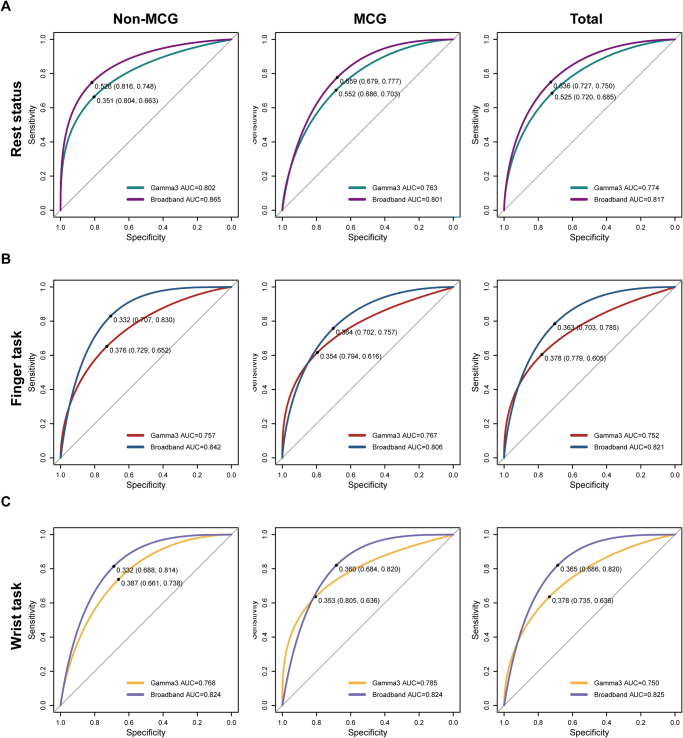
Performance of ECoG electrical activity in mapping the hand motor cortex. The AUC values display ECoG Gamma3 and broadband power during rest (A), finger movements (B), and wrist tasks (C) across different groups. Notably, the resting-state signals in the non-MCG group exhibit the most robust mapping for the M1 hand area, with optimal performance observed in the broadband frequency. In this group, the predictive efficacy for the finger task surpasses that of the wrist task, while the MCG group shows the opposite pattern.

**Figure 6.**
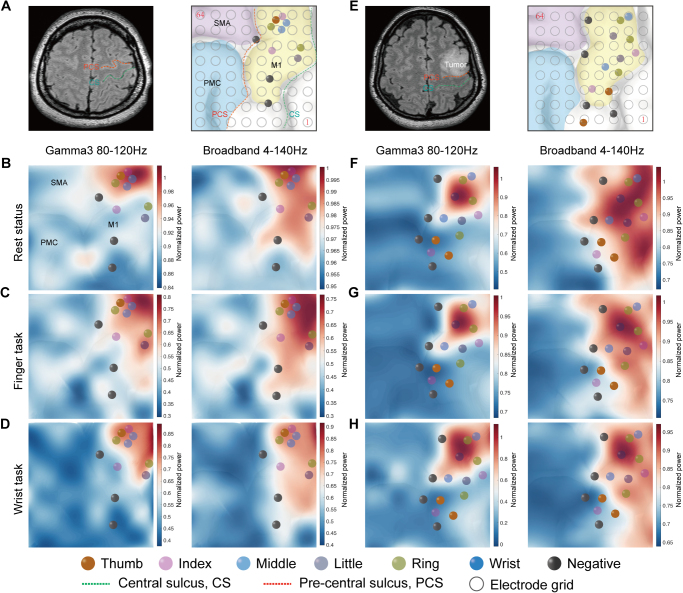
In Case 1 of the non-MCG group, the patient is diagnosed with a glioma in the temporal-insular lobe, while the M1 cortex remains unaffected (A, left). Stimulation response sites within the M1 hand area, highlighted in yellow, are observed (A, right), and the electrode grid partially covers this area. Electrical activity during the resting state (B), finger movements (C), and wrist tasks (D) effectively contributes to the functional mapping. Broadband power across various conditions shows a broader spatial distribution, whereas Gamma3 high-power activity is more localized. The relationship between ECoG activity mapping and stimulation localization is also examined in Case 2 of the MCG group, leading to similar conclusions as those observed in the non-MCG group (E-H). PCS, precentral sulcus; CS, central sulcus; SMA, supplementary motor area; PMA, pre-motor area; M1, primary motor cortex.

We applied the contour function with a parameter setting of 1000 to generate a heatmap that visualizes the spatial distribution of the ECoG power. The relationship between ECoG power and stimulation responses was evaluated by the AUC and metrics, including sensitivity, specificity, accuracy, positive predictive value (PPV), negative predictive value (NPV), and the Youden index (Fig. 5). Two representative cases further illustrated the clinical application of ECoG power feature mapping (Fig. 6).

## Statistical analyses

Data analysis and visualization were performed with R software (R Foundation, Vienna, Austria, version 4.1.2) and Python (version 3.7). Categorical data were analyzed with either the chi-squared test or Fisher’s exact test. The Mann–Whitney U test was employed to compare two independent samples. Dunn’s test was applied for nonparametric comparisons among multiple independent samples that were not normally distributed. Analysis of variance was used to identify significant differences across groups, with Tukey’s test for post hoc analysis if significant differences were found. All tests were two-sided, with a *P*-value of less than 0.05 indicating statistical significance.

## Results

### Participant characteristics

The non-MCG group included 12 patients with gliomas in the frontal or temporal lobe, sparing motor regions, while the MCG group consisted of 15 patients with gliomas infiltrating the motor cortex (SMA, PMC, and M1). No statistically significant differences were found in demographic characteristics, pathological features, or perioperative functional assessments (*P* > 0.05). None of the participants exhibited any significant preoperative clinical symptoms (Table 1).

**Table 1:** Demographic characteristics.

Variables	Non-MCG (*N* = 12)	MCG (*N* = 15)	*P* value
Age, years	37.1 ± 12.1	43.1 ± 12.3	0.221
Male, *n* (%)	6 (50.0)	10 (66.7)	0.450
Right-handed, *n* (%)	11 (91.7)	14 (93.3)	1.000
Education, years	13.1 ± 2.6	10.6 ± 4.1	0.072
Functional evaluation			
MoCA score	24.9 ± 3.2	23.9 ± 4.1	0.422
MMSE score	28.0 ± 2.1	28.5 ± 1.9	0.510
Fugl-Meyer score	99.9 ± 0.2	99.8 ± 0.3	1.000
Fugl-Meyer upper limb score	64.5 ± 0.8	64.3 ± 1.0	0.954
Tumor location, *n* (%)			0.007
Prefrontal lobe	3 (25.0)	0	
PMC	0	4 (26.6)	
SMA	0	5 (33.3)	
M1	0	1 (6.7)	
Temporal-insular	3 (25.0)	0	
Multiple lobes	6 (50.0)	5 (33.3)	
Tumor volume (ml)	48.9 (36.7, 66.2)	47.2 (29.8, 58.6)	0.558
EOR (%)			1.000
GTR/NTR	9 (75.0)	11 (73.3)	
PTR	3 (25.0)	4 (26.7)	
Histological, WHO grade, *n* (%)			0.351
Oligodendroglioma, 2	4 (33.3)	6 (40.0)	
Oligodendroglioma, 3	0	2 (13.3)	
Astrocytoma, 2	3 (25.0)	3 (20.0)	
Astrocytoma, 3	2 (16.7)	1 (6.7)	
Glioblastoma, 4	3 (25.0)	2 (13.3)	
IDH status, *n* (%)			0.628
Wild type	9 (75.0)	13 (86.7)	
Mutation	3 (25.0)	2 (13.3)	
Initial symptom, *n* (%)			0.963
Seizure	5 (41.7)	6 (40.0)	
Dizziness	1 (8.3)	2 (13.3)	
Headache	2 (16.7)	1 (6.7)	
Limb fatigue	1 (8.3)	2 (13.3)	
Limb numbness	2 (16.7)	2 (13.3)	
Speech deficits	2 (16.7)	3 (20.0)	
Post-surgery 1 week, *n* (%)			
Speech impairment	1 (8.3)	3 (20.0)	0.605
Muscle strength 0–2 grade	2 (16.7)	1 (6.7)	0.569
Post-surgery 1 month, *n* (%)			
Speech impairment	0	1 (6.7)	1.000
Muscle strength 0–2 grade	1 (8.3)	1 (6.7)	1.000

The extent of tumor resection (EOR) was categorized as follows: gross total resection (GTR), indicating complete tumor removal with no residual tissue; near-total resection (NTR), involving removal of more than 90% of the tumor; subtotal resection (STR), referring to the removal of 80–90% of the tumor; and partial resection (PR), involving excision of less than 80% of the tumor.

### Motor cortex glioma chaotic spatial distribution for hand activity

In the non-MCG group, tumors were primarily located in the temporal-insular and prefrontal lobes (Supplementary Figure 2A. link: http://links.lww.com/JS9/D903), while in the MCG group, they were concentrated in the SMA, PMC, and M1 (Fig. 2A left), as depicted by a KED heatmap. The distribution of positive sites in the two groups revealed that 76 positive sites in the non-MCG group were relatively localized, whereas 95 positive sites in the MCG group were more widely dispersed. Negative sites in both groups were also marked. All of these sites within the M1 hand cortex were registered to the MNI template (Supplementary Figure 2B and C. link: http://links.lww.com/JS9/D903).

In the Non-MCG group, individual fingers had specific locations, although these areas were not entirely isolated, displaying mild overlap on density heatmaps (Fig. 2A middle). Conversely, the MCG group’s cortical representations lacked distinct dispersion for individual fingers and had a markedly chaotic spatial arrangement (Fig. 2A right). Comparing the distribution of activated regions for identical fingers between both groups, cortical activation in the MCG group was more dispersed and followed different distribution patterns (Fig. 2B). The frequency of passive finger movements induced by stimulation varied between the two groups: the non-MCG group predominantly involved the thumb and index finger, while the MCG group more frequently engaged the ring and little fingers (Fig. 2C). The centroids of the Gaussian kernel density mapping for hand activity on the MNI template displayed a canonical spatial arrangement in the non-MCG group, exhibiting a linear relationship (*P* = 0.011). Conversely, this pattern was absent in the MCG group (*P* = 0.092) (Fig. 2D).

To further quantify these changes, Euclidean distances between the positive sites and the anatomical landmarks were measured (Supplementary Figure 3A. link: http://links.lww.com/JS9/D903). In the non-MCG group, cortical activation for finger and wrist movements typically clustered near the precentral sulcus and sagittal fissure. In the MCG group, these sites were closer to the central sulcus and Sylvian fissure (Fig. 3). A line chart displayed the differences in distances in various directions for the same gesture between the two groups. In the MCG group, the spatial variation for little finger movements was most prominent in the vertical direction, while horizontal shifts for ring finger movements were most pronounced (Supplementary Figure 3B. link: http://links.lww.com/JS9/D903).

### ECoG power features elevation in the M1 hand area and positive sites

The ECoG power across various signal states was compared between the two groups based on electrodes covering different brain regions. The results indicated that ECoG power in the M1 hand area was significantly higher than in other regions (Fig. 4). Additionally, this study confirmed that ECoG power at stimulation-positive sites was significantly greater than at negative sites, regardless of group differences or signal states (Supplementary Figure 4. link: http://links.lww.com/JS9/D903).

### Performance of ECoG electrical activity in mapping the hand motor cortex

By comparing the ECoG power with stimulation responses, we explored the effectiveness of cortical electrical activity in mapping the hand motor area (Fig. 5) (Table 2). Specifically, during resting status, the non-MCG group showed that the Gamma3 (80–120 Hz) band had an AUC of 0.802 (95% CI = 0.729–0.875), with a sensitivity of 0.744 and a specificity of 0.781. In contrast, the MCG group had an AUC of 0.763 (95% CI = 0.721–0.855), a sensitivity of 0.753, and a specificity of 0.823. For the broadband range (4–140 Hz), the non-MCG group achieved an AUC of 0.865 (95% CI = 0.804–0.926), a sensitivity of 0.732, and a specificity of 0.820, outperforming the MCG group, which had an AUC of 0.801 (95% CI = 0.766–0.899), a sensitivity of 0.814, and a specificity of 0.833.

**Table 2:** Performance of ECoG electrical activity in mapping the hand motor cortex.

Group	Signal	Frequency band	AUC	95%.CI	Sensitivity	Specificity	Accuracy	PPV	NPV	Youden index
Non-MCG	Resting-status	Gamma3	0.802	0.729–0.875	0.744	0.781	0.757	0.853	0.639	0.524
		Broadband	0.865	0.804–0.926	0.732	0.820	0.801	0.94	0.694	0.652
MCG	Resting-status	Gamma3	0.763	0.721–0.855	0.753	0.823	0.775	0.904	0.602	0.577
		Broadband	0.801	0.766–0.899	0.814	0.833	0.816	0.910	0.667	0.637
Total	Resting-status	Gamma3	0.774	0.737–0.835	0.623	0.864	0.703	0.902	0.534	0.487
		Broadband	0.817	0.788–0.880	0.771	0.831	0.791	0.899	0.645	0.602
Non-MCG	Finger task	Gamma3	0.757	0.687–0.852	0.661	0.848	0.779	0.717	0.811	0.509
		Broadband	0.842	0.781–0.924	0.743	0.907	0.847	0.822	0.857	0.647
MCG	Finger task	Gamma3	0.767	0.710–0.844	0.741	0.759	0.746	0.877	0.557	0.500
		Broadband	0.806	0.751–0.888	0.904	0.707	0.845	0.88	0.759	0.610
Total	Finger task	Gamma3	0.752	0.715–0.820	0.760	0.722	0.748	0.848	0.595	0.482
		Broadband	0.821	0.785–0.884	0.887	0.741	0.839	0.875	0.762	0.628
Non-MCG	Wrist task	Gamma3	0.768	0.686–0.866	0.860	0.720	0.809	0.841	0.750	0.580
		Broadband	0.824	0.758–0.909	0.895	0.732	0.831	0.846	0.800	0.616
MCG	Wrist task	Gamma3	0.785	0.719–0.849	0.718	0.740	0.725	0.866	0.531	0.459
		Broadband	0.824	0.753–0.890	0.889	0.656	0.819	0.857	0.717	0.545
Total	Wrist task	Gamma3	0.750	0.700–0.808	0.746	0.657	0.717	0.820	0.560	0.404
		Broadband	0.825	0.777–0.878	0.873	0.704	0.818	0.858	0.731	0.577

During finger movement tasks, the non-MCG group in Gamma3 band yielded an AUC of 0.757 (95% CI = 0.687–0.852), with a sensitivity of 0.661 and a specificity of 0.848. Meanwhile, the MCG group showed an AUC of 0.767 (95% CI = 0.710–0.844), with a sensitivity of 0.741 and a specificity of 0.759. The broadband range for the non-MCG group displayed an AUC of 0.842 (95% CI = 0.781–0.924), a sensitivity of 0.743, and a specificity of 0.907; while for the MCG group, the AUC was 0.806 (95% CI = 0.751–0.888), with a sensitivity of 0.904 and a specificity of 0.707.

During wrist movement tasks, the Gamma3 band in the non-MCG group had an AUC of 0.768 (95% CI = 0.686–0.866), with a sensitivity of 0.860 and a specificity of 0.720. The MCG group achieved an AUC of 0.785 (95% CI = 0.719–0.849), with a sensitivity of 0.718 and a specificity of 0.740. In terms of broadband, the non-MCG group reached an AUC of 0.824 (95% CI = 0.758–0.909), with a sensitivity of 0.895 and a specificity of 0.732, while the MCG group also achieved the same AUC of 0.824 (95% CI = 0.753–0.890), with a sensitivity of 0.889 and a specificity of 0.656.

### Clinical scenario for ECoG activity mapping M1 hand area

Two representative examples were examined to elucidate ECoG mapping the M1 hand area. In Case 1 of the non-MCG group, stimulation response sites within the M1 hand area were observed, and the electrode grid partially covered this area (Fig. 6A). The power in the M1 hand area was higher than that in other regions, with stimulation-positive sites primarily concentrated in high-power areas, while negative sites were mainly found in lower power regions. Broadband power across various states exhibited a broader distribution, whereas Gamma3 high power activity was more focused, consistent with previous neurocognitive findings (Fig. 6B–D). We also examined the relationship between ECoG mapping and stimulation localization in Case 2 of the MCG group, resulting in conclusions similar to those observed in the non-MCG group (Fig. 6E–H).

## Discussion

The omega-shaped region of the precentral gyrus, typically regarded as an anatomical marker for the hand motor cortex, may be reshaped by compression and infiltration from lesions. These alterations could result in a shift of the hand motor cortex from Brodmann area 4 to other cytoarchitectonic regions within the neocortex^[32,33]^. The detailed topography of our findings concerning hand activity in the non-MCG group aligns well with previous research^[34]^. In contrast, this spatial distribution tends to concentrate near the central sulcus and Sylvian fissure in the MCG group. Regions with diffuse overlap and shorter distances between adjacent digits exhibit lower activation strength and higher sensitivities to stimulation than those more specialized for individual fingers^[33,35]^. Variations in structure and neural activity, particularly in eloquent gliomas with ambiguous lesion boundaries, may increase the risk of neurological sequelae when DCS mapping is frequently employed.

High-frequency oscillations (>35 Hz) provide more specific signals and exhibit greater concordance with functional anatomy and DCS mapping than alpha and beta frequencies^[36,37]^. Increased cognitive effort induces high gamma (70–250 Hz) power modulations in the tumor-infiltrated cortex, indicating that these regions remain functional for complex executive tasks^[38]^. Task-related ECoG mapping in motor regions has demonstrated significantly higher sensitivity than fMRI, with values of 66.7% for ECoG and 52.6% for fMRI in patients with epilepsy (*P*<0.05)^[39]^. In this study, we evaluated the effectiveness of ECoG mapping across various motor tasks and explored that modifying the task paradigm in the lesion-affected motor cortex has minimal impact on mapping outcomes, and broadband oscillations associated with more complex cognitive activity may notably enhance predictive sensitivity but come at the expense of specificity^[40]^.

Complex visuomotor tasks that demand increased cognitive effort lead to greater gamma power fluctuations and distinct neural activity distributions in the normal cortex^[41,42]^. In this study, the wrist task paradigm activates the entire hand, potentially representing more complex motor execution compared to single-finger tasks. Consequently, wrist movement in the non-MCG group had a superior predictive performance within the Gamma3 band. Alpha and beta oscillations are the dominant neural components within the broadband, and event-related spectral power perturbations in the low-frequency band (<35 Hz) exhibit relatively mild alterations in glioma patients^[26,43,44]^. These factors may explain the observation that, during simple actions such as single-finger movements, the broadband range in the non-MCG group can evoke distinct patterns of neural activity in the motor cortex, ultimately achieving better mapping results. However, the variability in neural temporal-spatial dynamics caused by glioma infiltration in the motor cortex leads to better mapping performance for complex movements in both the Gamma3 and broadband frequencies, with broadband consistently yielding superior results^[33,35,45]^. This study is the first to emphasize the importance of complex movements and broadband frequencies for precise functional mapping in patients with affected motor cortex, contrasting sharply with the more localized distribution of Gamma3 activity.

Conducting specific motor tasks for ECoG mapping during AC poses significant challenges, especially for children and individuals with attention difficulties, which may limit the benefits from the procedure. Spontaneous cortical activity in a task-free (resting) state correlates with brain regions that are jointly active during tasks, with each component matching the spatial activity pattern observed during task performance^[40,46]^. Additionally, resting-state fMRI demonstrated a sensitivity of 90.91% and a specificity of 89.41% in localizing the hand motor area, whereas task-based fMRI exhibited a sensitivity of 78.57% and a specificity of 84.76%^[47,48]^. Moreover, resting-state networks can aid in mapping sensorimotor areas, with systematic seed selection in patients under anesthesia and those awake, yielding sensitivities of 78% and 83% and specificities of 67% and 60%, respectively. Nevertheless, the efficacy of ECoG functional mapping during resting state among patient with motor cortex gliomas remains uncertain. Our findings enhance this area of research by confirming that its performance closely mirrors the activation patterns associated with finger and wrist movements.

Despite our findings showing a strong correlation between ECoG and DCS mapping, ECoG mapping is not currently recommended for routine neurosurgical practice due to insufficient evidence to support its reliability. The spatial distribution of ECoG high-power activity and DCS-positive sites exhibited significant variability among individuals^[49,50]^. Our findings support the prevailing assumption that cortical mapping methods, such as fMRI, PET, EEG, and MEG, tend to identify larger activation areas than those localization by DCS^[51]^. Similar to other activation-based methods, ECoG mapping requires setting thresholds, either arbitrarily or empirically. Adjusting these thresholds across various frequency bands leads to corresponding variations in sensitivity and specificity^[37,52]^. Although the performance of high gamma mapping could potentially be enhanced by incorporating additional frequency bands, broadband activation-based ECoG mapping can identify cortical regions involved in processing that are not critical for functional activity^[18]^.

Despite the rigorous design of this study and the objectivity of its conclusions, several limitations need to be addressed. First, the size of the craniotomy limited electrode placement, preventing full coverage of the primary motor cortex and other regions involved in motor circuits, such as the somatosensory cortex and superior parietal lobule. These regions are essential for gesture encoding and information transmission. Second, the diverse effects of anesthetic agents, particularly propofol and dexmedetomidine, on neural activity must be taken into account^[53-55]^. It is essential to objectively evaluate the impact of these anesthetics on neural dynamics and to monitor the pharmacokinetics to minimize any influence on ECoG recordings. Third, DCS typically produces all-or-none results, and the number of electrode sites showing ECoG activity surpasses those tested with DCS. This leaves many high-power ECoG sites unvalidated, potentially affecting results in ways that are not yet completely understood. Fourth, previous studies indicate that the DCS mapping technique may overestimate functionally critical areas of the cortex while underestimating safe resection zones^[18,56]^. Consequently, the validation of ECoG mapping should focus not only on comparisons with DCS but also on assessing patients’ postoperative motor function. Collectively, future research should aim to increase the sample size, enhance signal quality, implement high-density electrode grids to maximize coverage of targeted brain regions, extract temporal-spatial features, and incorporate machine learning algorithms to optimize mapping performance.

## Conclusions

This study examined the disruptions in hand representation caused by motor cortex glioma, complicating functional mapping with intraoperative anatomical markers and the DCS technique. As a reliable alternative, ECoG-based procedures can enhance DCS mapping by decreasing the frequency of stimulation, potentially minimizing the risks of functional sequelae while optimizing tumor resection and preserving neurological function.

## Data Availability

The data supporting the findings of this study are not publicly available as they may compromise the privacy and consent of research participants. Anyone seeking access to the research-related data for validation or further study is required to request permission from the corresponding authors. Upon approval, the data will be made available to the authorized parties.

## Research registration unique identifying number (UIN)

ChiCTR2300074611.
